# The impact of rheumatoid arthritis on perioperative complications and hospitalization costs of lumbar fusion: a national analysis

**DOI:** 10.3389/fsurg.2025.1562714

**Published:** 2025-09-03

**Authors:** Lulu Ye, Wenyan Geng, Jian Wang, Qiuyuan Tang, Qinfeng Yang, Qiongxiao Wang, Gang Fang, Yuhang Chen, Yuzhou Pang

**Affiliations:** ^1^ Zhuang Medical College, Guangxi University of Chinese Medicine, Nanning, Guangxi, China; ^2^ Department of Blood Transfusion, Third Affiliated Hospital of Guangzhou Medical University, Guangzhou, Guangdong, China; ^3^ Division of Orthopaedic Surgery, Department of Orthopaedics, Nanfang Hospital of Southern Medical University, Guangzhou, Guangdong, China; ^4^ Department of Orthopedic Surgery, The First People’s Hospital of Foshan, Foshan, Guangdong, China

**Keywords:** lumbar fusion, rheumatoid arthritis, complications, hospitalization costs, the nationwide inpatient sample database

## Abstract

**Background:**

Several studies have evaluated the influence of rheumatoid arthritis (RA) on postoperative results following lumbar fusion. Nonetheless, there is a lack of national-level data on the hospitalization costs and perioperative complications associated with RA patients who underwent lumbar fusion.

**Methods:**

The Nationwide Inpatient Sample (NIS) database was analyzed from 2011 to 2019, focusing on individuals who received lumbar fusion procedures. Our retrospective study included 282,339 patients identified based on the International Classification of Diseases, Ninth and Tenth Editions (ICD-9/10). A comparative analysis was conducted using the outcomes of 8,993 patients with RA and 273,346 non-RA (NRA) patients. Propensity score matching was performed for the RA vs. NRA patients (1:2) in each procedure group to control for confounding demographic variables.

**Results:**

Patients with RA were notably older on average (64 years vs. 60 years in NRA, *P* < 0.001) and had a higher proportion of females (*P* < 0.001). The RA cohort also experienced significantly longer hospital stays (*P* < 0.001) and higher hospital charges. Regarding perioperative complications, individuals with RA were significantly more likely to develop postoperative anemia (RA 22.2%vs. NRA 18.2%, *P* < 0.001) and require blood transfusions (RA 11.7% vs. NRA 9.7%, *P* < 0.001). No significant differences were observed in other recorded inpatient complications between RA and NRA patients.

**Conclusion:**

Despite longer hospital stays and increased hospital charges, patients with RA did not show an increased risk of most in-hospital complications associated with lumbar fusion during the perioperative phase in the United States, except for postoperative anemia and the requirement for blood transfusions.

## 1 Introduction

The incidence of spinal surgeries has been on the rise, driven by advances in implants, evolving techniques, and expanded indications for spine surgery (1). The United States leads globally in the frequency of spinal surgeries performed yearly (2). The lumbar region, bearing much of the body's weight and facilitating movement, is particularly prone to degenerative changes and injury. Consequently, lumbar surgeries constitute a significant proportion of all spinal surgical procedures. Moreover, a study by Hunter et al. (3) reported a substantial increase in the prevalence of rheumatoid arthritis (RA) in the United States. Given its growing incidence, spine surgeons and other healthcare providers are likely encounter RA patients more frequently throughout their careers.

RA is an autoimmune inflammatory condition characterized by polyarthritis affecting both small and large joints. Studies have highlighted the prevalence of lumbar spine involvement in RA patients. A cross-sectional analysis indicated that 24% of RA patients experience lumbar discomfort (4), and a significantly higher incidence of lumbar lesions (45%) is observed in patients with RA duration exceeding ten years (5). As musculoskeletal disabilities worsen, surgical interventions often become necessary, including lumbar fusion. Compared to laminectomy or discectomy, lumbar fusion is a more intricate and comprehensive surgical approach (6). Of note, fusion procedures are associated with a higher risk of complications due to extensive tissue manipulation, longer operative times, increased risk of hemorrhage, and elevated mortality rates compared to many other spine surgeries (7). Furthermore, RA patients are more likely to have comorbidities and often require immunomodulatory medications such as biologics and Janus kinase inhibitors, which can increase the risk of adverse outcomes. Nevertheless, lumbar fusion is still considered a viable treatment option for RA patients. It is crucial to thoroughly assess the impact of the disease on these patients, especially in light of the increased risks.

To our knowledge, no nationwide, population-based studies have analyzed in-hospital complications and medical expenses in RA patients receiving lumbar fusion intervention. With recent changes in the healthcare landscape in the United States, it is increasingly critical to assess the economic impact on medical practice, particularly in patients with complicated conditions (8). To that end, our study aimed to evaluate perioperative outcomes and hospitalization costs associated with lumbar spinal fusion in individuals with RA compared to those without, using data obtained from the Nationwide Inpatient Sample (NIS) dataset. Specifically, the study encompassed the following: (1) characterizing the demographic profile of patients; (2) identifying perioperative surgical and medical complications; (3) determining the lengths of stay (LOS); and (4) calculating overall hospitalization expenses for individuals with RA who underwent lumbar fusion surgery.

## 2 Materials and methods

### 2.1 Data source

This study utilized data obtained from NIS, the most extensive American inpatient care database, capturing approximately 8 million hospital admissions each year across about 1,000 hospitals. The dataset includes comprehensive patient demographics, covering those enrolled in Medicare, Medicaid, private insurance, and those uninsured. Moreover, it encompasses diagnostic and procedural codes as per the International Classification of Diseases, Ninth and Tenth Revisions, Clinical Modification (ICD-9/10-CM), along with insurance details, hospital-specific data, overall hospitalization expenses, LOS, and patient discharge statuses. The outcomes derived from this database are representative of RA patients undergoing lumbar fusion across the United States. Ethical approval was waived by the institutional review board due to the use of anonymized, publicly accessible data.

### 2.2 Data collection

Patients who underwent lumbar fusion between 2011 and 2019 were identified using the ICD-9-CM and ICD-10-CM diagnosis and procedure codes (8,105/8,106/8,108) (*n* = 337,812). Patients were categorized into two groups: those with RA and those without RA (ICD-9: 714.x; ICD-10: M05, M06). Primary demographic factors, including gender, age group at admission, race, and postoperative complications, were investigated across both cohorts using ICD-9-CM and ICD-10-CM diagnosis codes. The term “any complication” was used to denote the occurrence of one or more surgical or medical complications. The spectrum of medical complications included postoperative anemia, thrombocytopenia, acute renal failure, acute myocardial infarction, pneumonia, pulmonary embolism, urinary tract infection, deep vein thrombosis, sepsis/septicemia, postoperative shock, urinary retention, pain, nausea and vomiting, and arrhythmia. Surgical complications included blood transfusion, hemorrhage, seroma, hematoma, wound infection, wound dehiscence, and bronchial spasm. Length of stay was calculated as the period from the admission date to the discharge date, and overall hospital charges were also gathered from the database.

**Figure 1:**
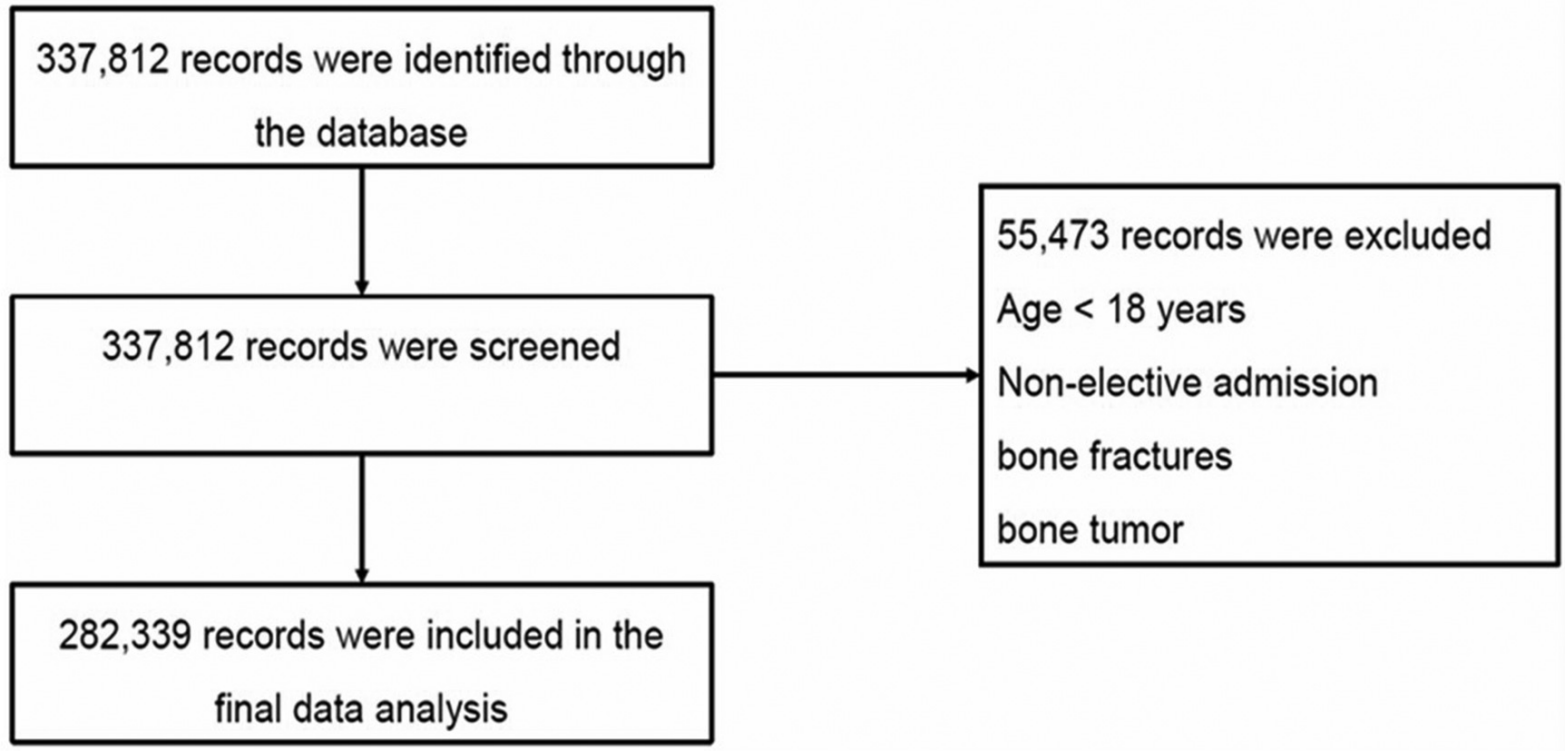
Flow diagram of the study workflow and patient selection process.

As shown in Figure 1, patients were excluded (*n* = 55,473) if they were under18 years old, required emergency treatment, or those that were admitted through the emergency department, had bone fractures (8,054/8,055/8,058/8,059/8,064/8,065/8,068/8,069) or bone tumors (1,702/2,132/C412/D166). A total of 8,993 RA patients who underwent lumbar fusion from 2011 to 2019 were eventually selected (Table 1).

**Table 1:** Demographics of lumbar fusion patients with and without rheumatoid arthritis.

Variable	Rheumatoid arthritis	No rheumatoid arthritis	*P*
*N*	8,993	273,346	–
Total comorbidity rate, %	3.2%		
Age in years, mean	64	60	<0.001
Age distribution, *n* (%)			
18–44	283 (3.1%)	34,371 (12.6%)	<0.001
45–64	3,929 (43.7%)	121,705 (44.5%)	
65–74	3,415 (38.0%)	81,521 (29.8%)	
≥75	1,366 (15.2%)	35,749 (13.1%)	
Sex, *n* (%)			
Male	2,297 (25.5%)	121,775 (44.5%)	<0.001
Female	6,696 (74.5%)	151,571 (55.5%)	
Race, *n* (%)			
White	7,116 (82.9%)	216,251 (79.1%)	0.103
Black	689 (8%)	19,558 (7.2%)	
Hispanic	466 (5.4%)	14,245 (5.2%)	
Asian or Pacific Islander	90 (1.0%)	3,003 (1.1%)	
Native American	47 (0.5%)	6,144 (2.2%)	
Other	176 (2.1%)	13,018 (4.8%)	
Insurance, *n* (%)			
Medicare	5,483 (61.0%)	126,362 (46.2%)	<0.001
Medicaid	391 (4.3%)	16,031 (5.9%)	
Private insurance	2,813 (31.3%)	107,440 (39.3%)	
Self-pay	32 (0.4%)	1,689 (0.6%)	
No charge	4 (0.0%)	149 (0.1%)	
Other	270 (3.0%)	21,675 (7.9%)	

### 2.3 Propensity score matching

A propensity score matching analysis was conducted to control for the influence of demographic variables that could confound the results. After matching, the ratio of RA to NRA patients was adjusted to 1:2, resulting in a cohort of 8,992 RA patients and 17,984 NRA patients (Table 2). Random patient selection was implemented in this study, resulting in two cohorts that exhibited matching distributions across age, gender, race, and hospital discharge.

**Table 2:** Demographics of rheumatoid arthritis and the matched cohort of lumbar fusion patients.

Variable	Rheumatoid arthritis	Matched controls	*P*
N	8,992	17,984	
Age-group, *n* (%)			
18–44	283 (3.1%)	511 (2.8%)	0.361
45–64	3,928 (43.7%)	8,004 (44.5%)	
65–74	3,415 (38.0%)	6,742 (37.5%)	
≥75	1,366 (15.2%)	2,727 (15.2%)	
Sex, *n* (%)			
Male	2,297 (25.5%)	4,612 (25.6%)	0.859
Female	6,695 (74.5%)	13,372 (74.4%)	
Race, *n* (%)			
White	7,116 (79.1%)	14,184 (78.9%)	0.946
Black	689 (7.7%)	1,422 (7.9%)	
Hispanic	466 (5.2%)	945 (5.3%)	
Asian or Pacific Islander	90 (1.0%)	192 (1.1%)	
Native American	46 (0.5%)	83 (0.5%)	
Other	585 (6.5%)	1,158 (6.4%)	
Insurance, *n* (%)			
1 = Medicare	5,482 (61.0%)	10,799 (60.0%)	0.652
2 = Medicaid	391 (4.3%)	769 (4.3%)	
3 = Private insurance	2,813 (31.3%)	5,821 (32.4%)	
4 = Self-pay	32 (0.4%)	60 (0.3%)	
5 = No charge	4 (0.0%)	8 (0.0%)	
6 = Other	270 (3.0%)	527 (2.9%)	

### 2.4 Data analysis

All statistical procedures were performed using SPSS statistics software, version 25.0 (IBM Corporation, Armonk, NY, USA). We evaluated the incidence of surgical complications, the frequency of post-surgical adverse events, mortality rates, LOS, and overall hospitalization costs. To mitigate the influence of potential confounding variables in the comparative analysis of perioperative complications, mortality rates, LOS, and overall hospitalization costs, a 1:2 propensity score matching approach was adopted, calibrating for differences in age, sex, ethnicity, elixhauser comorbidity index (ECI), and insurance classification. A multivariable logistic regression analysis was employed to determine the odds ratios (ORs) for surgical and postoperative complications occurring in the RA patient group vs. the matched control group without RA. We conducted a linear regression analysis to derive parameter estimates for average LOS and total hospitalization expenses to examine their combined impact on RA patients. The linear regression results are presented as percentage variations, and the ORs with corresponding 95% confidence intervals (CIs) were computed using the formula (eb−1) × 100, in which b denotes the logarithm of the estimated parameters of the dependent variable.

## 3 Results

### 3.1 Prevalence of RA in the lumbar interbody fusion population

From 2011 to 2019, a total of 282,339 cases undergoing lumbar fusion were identified within the NIS database. During this period, the prevalence of RA among these cases increased progressively from 2.7% to 3.8%, with an overall nine-year prevalence of 3.2% among patients who underwent lumbar fusion.

### 3.2 Characteristics of RA patients

The comparison of demographic profiles revealed substantial differences between individuals with RA and those without, particularly in terms of age, sex, and type of primary insurance (Table 1). Individuals with RA who underwent lumbar fusion were significantly older (mean age, 64 years vs. 60 years, *P* < 0.001), of the female gender (74.5% vs. 55.5%, *P* < 0.001), and were more likely to be enrolled in Medicare (61.0% vs. 46.2%, *P* < 0.001). Moreover, the prevalence rate was 10.3% greater among individuals aged 65 and above (53.2% vs. 42.9%, *P* < 0.001).

### 3.3 Perioperative surgical and medical complications

A statistically significant variance was detected in the occurrence of overall complications, surgical-specific complications, and medical-specific complications among individuals with RA compared to the control group (Table 3). Analysis of specific medical complications revealed that patients with RA faced a 1.22-fold elevated risk of postoperative acute anemia relative to the matched control group (OR = 1.224; 95% CI = 1.146–1.307; *P* < 0.001) (Table 4). Regarding surgical complications, the RA patient group exhibited a higher requirement for blood transfusions (OR = 1.170; 95% CI = 1.076–1.272; *P* < 0.001) compared to the matched cohort (Table 5).

**Table 3:** Perioperative complications in patients undergoing lumbar fusion with or without rheumatoid arthritis.

Parameter	Rheumatoid arthritis^a^	Matched controls	*P*
Any complication	1.161 (1.097–1.229)	1.000	<0.001
Any medical complication	1.149 (1.084–1.218)	1.000	<0.001
Any surgical complication	1.165 (1.074–1.264)	1.000	<0.001

^a^ Data are presented as odds ratio and (95% confidence intervals).

**Table 4:** Medical complications in patients undergoing lumbar fusion with and without rheumatoid arthritis.

Parameter	Univariate analysis			Multivariate logistic regression	
	Rheumatoid arthritis^a^	Matched controls	*P*	OR (95% CI)	*P*
Postoperative anemia	1,992 (22.2%)	3,282 (18.2%)	<0.001	1.224 (1.146–1.307)	<0.001
Thrombocytopenia	229 (2.5%)	394 (2.2%)	0.067	0.924 (0.628–1.358)	0.687
Acute renal failure	264 (2.9%)	462 (2.6%)	0.079	1.003 (0.852–1.180)	0.975
Acute myocardial infarction	48 (0.5%)	93 (0.5%)	0.858	0.906 (0.633–1.296)	0.589
Pneumonia	94 (1.0%)	181 (1.0%)	0.764	0.885 (0.683–1.147)	0.357
Pulmonary embolism	27 (0.3%)	59 (0.3%)	0.703	0.849 (0.518–1.389)	0.514
Urinary tract infection	270 (3.0%)	445 (2.5%)	0.011	1.160 (0.992–1.356)	0.062
Deep vein thrombosis	47 (0.5%)	63 (0.4%)	0.036	1.374 (0.934–2.022)	0.106
Sepsis/Septicemia	33 (0.4%)	68 (0.4%)	0.888	0.836 (0.542–1.290)	0.419
Postoperative shock	22 (0.2%)	42 (0.2%)	0.860	0.935 (0.547–1.599)	0.806
Urinary retention	336 (3.7%)	764 (4.2%)	0.045	0.837 (0.733–0.955)	0.008
Pain	356 (4.0%)	615 (3.4%)	0.025	1.115 (0.974–1.276)	0.115
Nausea and vomiting	268 (3.0%)	477 (2.7%)	0.121	1.120 (0.961–1.305)	0.148
Arrhythmia	29 (0.3%)	44 (0.2%)	0.246	1.238 (0.770–1.989)	0.378

OR, odds ratio; CI, confidence interval.

^a^ Cells with frequency <11 are suppressed due to the protection of patient privacy.

**Table 5:** Surgical complications in patients undergoing lumbar fusion with and without rheumatoid arthritis.

Parameter	Univariate analysis			Multivariate logistic regression	
	Rheumatoid arthritis^a^	Matched controls	*P*	OR (95% CI)	*P*
Blood transfusion	1,051 (11.7%)	1,738 (9.7%)	<0.001	1.170 (1.076–1.272)	<0.001
Hemorrhage/seroma/hematoma	70 (0.7%)	128 (0.8%)	0.545	1.094 (0.817–1.466)	0.545
Wound infection	21 (0.2%)	29 (0.2%)	0.193	1.409 (0.795–2.495)	0.240
Wound dehiscence	12 (0.1%)	31 (0.2%)	0.450	0.727 (0.370–1.426)	0.354
Bronchial spasm	2 (0.0%)	5 (0.0%)	1.000	0.765 (0.145–4.052)	0.753

^a^ Cells with frequency <11 are suppressed due to the protection of patient privacy.

### 3.4 Length of stay and total hospital charges

In terms of hospital LOS, individuals with RA had an average duration of 4.03 days, compared to 3.75 days for patients without RA (*P* < 0.001), with linear regression analysis showing that RA patients exhibited an increased LOS of 0.280 days (95% CI, 0.195–0.365, *P* < 0.001). When compared to the matched cohort, the presence of RA was associated with a notable increase in mean overall hospitalization expenses, exceeding by an average of $9,490.33 ($152,404.82 vs. $142,914.49, 95% CI, Ig 149,844.68 - Ig 154,964.96; *P* < 0.001) (Table 6).

**Table 6:** Hospital outcomes for patients undergoing lumbar fusion with and without rheumatoid arthritis.

Parameter	Rheumatoid arthritis	Matched controls	*P*
Lengths of stay (days)			
Mean (SD)	4.03 (3.25)	3.75 (3.60)	<0.001
Percentage difference (95% CI)	0.280 (0.195–0.365)	0.00	<0.001
Charges ($)			
Mean (SD)	152,404.82 (123,308.33)	142,914.49 (113,213.04)	<0.001
Percentage difference (95% CI)	9,490.33 (6,438.51–12,542.16)	0.00	<0.001
Mortality rate (%)			
Rate	0.10	0.10	0.899
Percentage difference (95% CI)	0.229 (0.160–0.297)	0.00	<0.001

OR, odds ratio; CI, confidence interval.

## 4 Discussion

The number of subjects included in this retrospective study (282,339) significantly exceeds any previously reported study focusing on perioperative outcomes associated with lumbar fusion. Therefore, our findings may provide a more comprehensive representation of patients' conditions, both with and without RA, and more accurately delineate potential differences in perioperative outcomes and hospitalization costs between these two cohorts. Our findings suggest the following: (1) The incidence of RA among individuals undergoing lumbar fusion increased continuously from 2.7% in 2011 to 3.8% in 2019; (2) Patients with RA faced similar risks of encountering in-hospital complications to those without RA, except for a higher risk of experiencing acute postoperative anemia and requiring blood transfusions; (3) Hospitalized RA patients had longer LOS and higher hospitalization costs.

In the present study, RA patients tended to be older and predominantly female, aligning with previous literature (9). We also found that RA patients undergoing lumbar fusion have an increased risk of developing acute postoperative anemia and are more likely to require blood transfusions, similar to findings in RA patients undergoing total knee and shoulder arthroplasty (10, 11). No definitive reason for this can be ascertained from the data in this study. It may, however, be associated with the high prevalence of anemia in these patients as reported by Ratnasamy PP et al. (9). It is widely known that anemia is frequently observed in RA and is often multifactorial. Moreover, numerous studies have confirmed that spine fusion surgery is linked to significant intraoperative blood loss (12, 13), with rates in adult spine surgery reaching as high as 50- 80% (14). In addition to visible blood loss from sources such as suction drainage, researchers are increasingly concerned with hidden blood loss (HBL) in RA patients. Smorgick et al. (15) highlighted the significance of HBL, which accounts for approximately 50% of TBL. Wen et al. (16) found that the average HBL following Posterior Lumbar Interbody Fusion (PLIF) surgery was around 588 ml, exceeding clinical expectations. The augmented intraoperative bleeding observed in RA patients may be attributable to the underlying pathological involvement of synovial inflammation in RA (17, 18).

On one hand, a well-documented complication in RA patients that has received extensive research attention is venous thromboembolic (VTE) disease (19). Autoimmune inflammatory events driven by RA are correlated with an enhanced susceptibility to VTE. However, the role of RA as a potential contributing factor in increasing patients' susceptibility to the development of postoperative VTE remains debatable. A systematic review indicates that there is no elevated risk of VTE in individuals with RA who undergo orthopedic surgery, a finding that aligns with our data (20). Similarly, the study by Gulati A et al. (21) concluded that RA does not confer an additional risk for the occurrence of postoperative VTE in surgical patients. An investigation assessing D-dimer levels found no difference in VTE susceptibility between RA and osteoarthritis patients after primary TKA (22), suggesting that RA may not predict VTE onset.

On the other hand, the increased likelihood of infection in RA patients undergoing orthopedic surgery remains an ongoing concern due to compromised immune responses associated with RA or side effects of immunosuppressive therapeutic regimens (23). Contrary to theoretical expectations of a higher infection rate, Chung et al. (24) revealed that, after adjusting for potential confounding variables, the risk of acute surgical site infections in TKA does not differ significantly between RA and OA patients. Similarly, Gulati A et al. (21) reported no significant difference in surgical site infections between RA and NRA patients undergoing surgery for lumbar spinal stenosis. In our study, postoperative infection incidences among both groups were comparable. Da Cunha et al. (25) concluded that RA does not constitute a significant risk factor for the development of perioperative infections following TKA. We propose that the lower incidence of surgical site infections may be attributable to the efficacy of standard antirheumatic treatments and comprehensive perioperative care. Nevertheless, these lower values may merely reflect the incidence of infection during hospitalization, and long-term follow-up may reveal a higher infection rate. Therefore, continued focus on monitoring long-term infection risks is essential for RA patients following lumbar fusion.

In general, despite a higher burden of medical comorbidities compared to NRA patients, RA patients appear to be less susceptible to additional medical complications (21, 26). For instance, cardiac event occurrence was lower among RA patients. Stundner et al. (26) attributed this decreased frequency of complications to several factors, notably the increased medical attention received by RA patients from various specialties leading up to and immediately following surgery. Our findings indicate that acute postoperative anemia and blood product transfusions were more commonly observed as perioperative adverse events in RA patients, whereas the incidence of other adverse events was comparable between the RA and NRA groups. This is consistent with earlier findings from research by Chu et al. (27) and Gulati et al. (21). The high incidence of spinal surgery in the United States and ongoing improvements in surgical techniques may have contributed to reduced complication risks, as evidenced in the lower complication rate observed among RA patients in this retrospective study. Moreover, our study's focus on elective primary lumbar fusion, excluding emergency surgeries for fractures and bone tumors, which are associated with more complex perioperative complications (28, 29), suggests effective preoperative and perioperative management for RA patients 6 undergoing elective lumbar fusion is essential.

In line with several previous studies (30, 31), our findings revealed an increase in LOS and hospital costs in the RA group vs. the NRA group. This is likely due to the comprehensive preoperative examinations and preparations required for RA patients, as well as the higher incidence of postoperative complications and associated increased treatment costs. Notably, our study identified a significantly higher risk of blood transfusion in the RA group. Similarly, a prior study using data from the American College of Surgeons National Surgical Quality Improvement Program (ACS-NSQIP) linked blood transfusions to extended postoperative LOS among patients undergoing primary posterior lumbar fusion (32). Stokes ME et al. (33) further suggest that postoperative blood transfusions may contribute to prolonged hospital stays and potentially increased hospitalization costs.

This study has five methodological limitations (34–36): (1) The sample derived from the US NIS database represents predominantly North American populations, whose demographic, socioeconomic, and insurance characteristics may limit the generalizability of findings to other healthcare systems. (2) Although we restricted the analysis to elective, primary, degenerative lumbar fusion, ICD-9/10 codes cannot reliably distinguish pathological subtypes such as isthmic vs. degenerative spondylolisthesis, introducing potential misclassification that may reduce internal validity. (3) The retrospective design lacks critical covariates—including RA disease activity [Disease Activity Score-28 (DAS28), C-reactive protein (CRP)/erythrocyte sedimentation rate (ESR)], disease-modifying antirheumatic drugs (DMARDs)/biologics exposure, and surgical technical details—thereby precluding etiological stratification; thus, residual confounding is inevitable and the study can only generate real-world evidence rather than establish causality. (4) Administrative ICD codes may incompletely capture complications, and the absence of chart review predisposes to under-reporting and misclassification bias. (5) The database captures in-hospital events only; post-discharge infections, thromboembolism, or readmissions are not recorded, potentially under estimating true 30–90-day complication rates, particularly in RA patients with prolonged immunosuppression. In summary, our findings provide macro-level risk signals within the current methodological constraints; future multicenter prospective cohorts that integrate granular rheumatologic and surgical data and stratify by pathological subtype are warranted to validate and refine these conclusions.

## 5 Conclusion

This study indicates an increased prevalence of RA patients undergoing lumbar fusion, with RA patients exhibiting a higher risk of postoperative anemia and blood transfusions than NRA patients. Additionally, RA patients experience a longer LOS and higher hospitalization costs. Healthcare providers should anticipate these complications to either prevent them or minimize their impact. Furthermore, in an era of healthcare system management with a focus on cost control, future research should aim to enhance the management of lumbar fusion for RA patients by targeting the prevention of complications, thereby improving patient outcomes and reducing hospitalization costs.

## Data Availability

The datasets presented in this study can be found in online repositories. The names of the repository/repositories and accession number(s) can be found below: The NIS database is a large publicly available all-payer inpatient care database in the United States and the direct web link to the database is https://www.ahrq.gov/data/hcup/index.html. Further inquiries can be directed to the corresponding author.
